# Obesity as a Potential Explanatory Factor in the Association Between Socioeconomic Position and Multidimensional Cardiometabolic Risk

**DOI:** 10.3390/medsci14030382

**Published:** 2026-07-09

**Authors:** María Teófila Vicente-Herrero, Ángel Arturo López González, Pedro J. Tárraga López, Carla Busquets-Cortés, Lluis Rodas Cañellas, José Ignacio Ramírez Manent

**Affiliations:** 1 ADEMA University School, University of the Balearic Islands, 07009 Palma, Spain; correoteo@gmail.com (M.T.V.-H.); ll.rodas@eua.edu.es (L.R.C.); joseignacio.ramirez@ibsalut.es (J.I.R.M.); 2 Faculty of Medicine of Castilla La Mancha, 02008 Albacete, Spain; pjtarraga@sescam.jccm.es

**Keywords:** obesity, cardiometabolic risk, insulin resistance, metabolic syndrome, fatty liver

## Abstract

Background. Cardiometabolic diseases are strongly socially patterned, with individuals of lower socioeconomic position experiencing a disproportionate burden of metabolic disorders. Obesity has been proposed as a potential explanatory factor that may partly account for the association between social disadvantage and adverse cardiometabolic profiles. This study aimed to evaluate whether obesity may partly explain the observed associations between socioeconomic position and multiple cardiometabolic outcomes. Methods. A cross-sectional study was conducted in 3108 working-age adults undergoing occupational health examinations in the Balearic Islands, Spain. Socioeconomic position was assessed using educational attainment and occupational category. Cardiometabolic outcomes included obesity, FLI-defined probable steatotic liver disease (FLI ≥ 60), insulin resistance, atherogenic risk, and metabolic syndrome. Multivariable logistic regression models with sequential adjustment were performed. Additional models evaluated attenuation after inclusion of obesity. Mediation analyses were conducted using bootstrapping methods. Results. A clear socioeconomic gradient was observed across all cardiometabolic outcomes, with higher prevalence among individuals with lower educational attainment (*p* for trend < 0.001). In fully adjusted models, lower educational level remained significantly associated with obesity (OR 1.61; 95% CI 1.18–2.21), whereas associations with the remaining cardiometabolic outcomes were substantially attenuated after adjustment. Inclusion of obesity reduced effect estimates for FLI-defined probable steatotic liver disease (FLI ≥ 60), insulin resistance, atherogenic risk, and metabolic syndrome by approximately 53–72%. Exploratory mediation analyses suggested that obesity was associated with approximately 28% attenuation of the association with estimated steatotic liver disease risk and 8% attenuation of the association with insulin resistance. Conclusions. Socioeconomic inequalities in cardiometabolic risk were evident across multiple metabolic domains. Obesity was associated with substantial attenuation of several downstream cardiometabolic associations and may represent an important explanatory factor associated with the observed socioeconomic differences in metabolic health, particularly for estimated steatotic liver disease risk. However, the magnitude of these associations varied across outcomes, and the cross-sectional design precludes causal interpretation. These findings support the importance of addressing obesity within its broader social context as a potential strategy to reduce metabolic health inequalities among employed working-age adults.

## 1. Introduction

Cardiometabolic diseases remain the leading cause of morbidity and mortality worldwide, accounting for a substantial proportion of global disability and healthcare burden [1,2,3,4,5]. Despite advances in prevention and clinical management, the prevalence of these disorders continues to rise across both high-income and transitioning populations, reflecting the complex interplay between biological, behavioural, and environmental determinants [6,7].

There is extensive evidence indicating that cardiometabolic health follows a clear socioeconomic gradient [8,9,10]. Individuals with lower socioeconomic position consistently exhibit higher prevalence of obesity, type 2 diabetes, and cardiovascular disease, as well as poorer metabolic profiles overall [11,12]. These inequalities persist across different healthcare systems and geographical contexts, suggesting that access to medical care alone is insufficient to eliminate disparities in health outcomes [13]. Educational attainment has been identified as one of the most robust indicators of socioeconomic position in epidemiological research because it reflects long-term access to resources, health literacy, and opportunities for adopting healthy behaviours [14,15,16,17,18]. Lower educational levels have been consistently associated with obesity, insulin resistance, and adverse metabolic profiles [19,20]. Although behavioural factors such as diet, physical activity, and smoking contribute to these associations, they do not fully explain the observed socioeconomic gradients [21,22], suggesting the involvement of broader structural and contextual determinants [23].

Obesity occupies a particularly important position within this framework. Excess adiposity is highly prevalent and is strongly associated with downstream metabolic disturbances, including insulin resistance, hepatic steatosis, systemic inflammation, and atherogenic dyslipidaemia [24,25]. From an epidemiological perspective, obesity is also socially patterned, with higher prevalence consistently observed among individuals with lower socioeconomic position [26,27]. This dual relationship, as both a correlate of social disadvantage and a marker of adverse metabolic health, supports the hypothesis that obesity may represent an important explanatory factor that could partly account for the association between socioeconomic position and broader cardiometabolic risk [28].

Cardiometabolic abnormalities frequently cluster within individuals, reflecting interconnected biological processes and shared upstream determinants [29,30,31]. This clustering has been associated with increased risk of cardiovascular events, type 2 diabetes, and premature mortality [32]. However, many epidemiological studies continue to evaluate isolated outcomes, which may underestimate the complexity of cardiometabolic dysfunction and limit the identification of key pathways underlying socioeconomic inequalities [33]. In addition, life-course socioeconomic exposures may contribute to long-term metabolic trajectories and persistent cardiometabolic inequalities [34,35,36,37].

Despite the growing literature on socioeconomic inequalities in cardiometabolic health, it remains unclear whether these associations operate independently across metabolic domains or are largely influenced by potential explanatory factors. Clarifying this issue may improve current conceptual models and help identify more effective prevention strategies [38,39].

Therefore, the present study aimed to examine the association between educational attainment, considered the primary indicator of socioeconomic position in the present analyses, and multiple cardiometabolic outcomes within a multidimensional framework. Occupational category was included as a complementary descriptive indicator of socioeconomic circumstances. In particular, we sought to evaluate whether obesity may partly explain the observed associations between socioeconomic disadvantage and downstream metabolic dysfunction and to quantify the extent to which adjustment for obesity attenuates these associations.

## 2. Methods

### 2.1. Study Design and Setting

A cross-sectional study was conducted using data from a cohort of working-age adults who underwent routine occupational health examinations in the Balearic Islands, Spain. These assessments were performed within workplace health surveillance programmes and included standardised collection of sociodemographic, anthropometric, clinical, and laboratory data. The study was conducted in accordance with the Declaration of Helsinki and approved by the Research Ethics Com-mittee of the Balearic Islands (protocol code IB 4383/20 PI; approval date: 6 October 2020). The study was designed and reported in accordance with the Strengthening the Reporting of Observational Studies in Epidemiology (STROBE) recommendations for observational research [38].

### 2.2. Study Population

**Figure 1:**
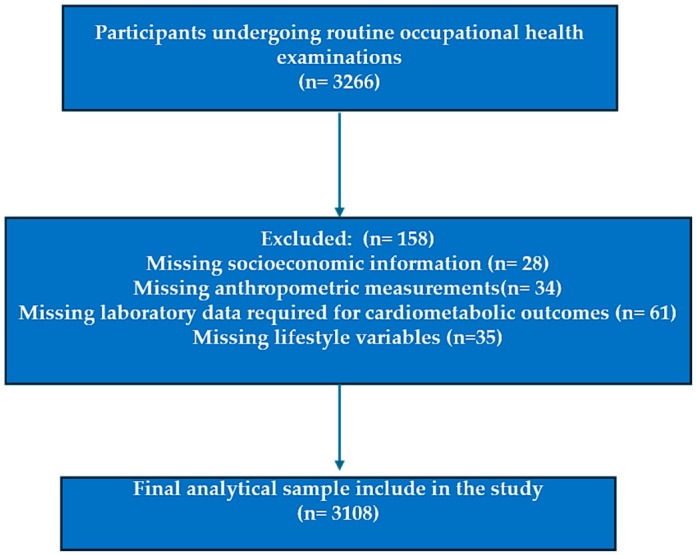
STROBE flow diagram.

Eligible participants were men and women aged 18 to 65 years with complete information on socioeconomic indicators, anthropometric measurements, lifestyle variables, and cardiometabolic outcomes. A total of 3266 individuals underwent routine occupational health examinations during the study period. Of these, 158 participants were excluded because of missing socioeconomic information (*n* = 28), missing anthropometric measurements (*n* = 34), missing laboratory data required for the definition of cardiometabolic outcomes (*n* = 61), or missing lifestyle variables, including smoking status, physical activity, and Mediterranean diet adherence (*n* = 35). The final analytical sample therefore comprised 3108 participants. The participant selection process is summarised in Figure 1.

### 2.3. Socioeconomic Variables

Socioeconomic position was assessed using two complementary indicators: educational attainment and occupational category. Educational level was classified into three ordinal categories according to the highest level completed: higher education, intermediate education, and primary education or no formal education. This categorisation was selected to capture long-term socioeconomic gradients in a manner consistent with previous analyses of this cohort. Because educational attainment represents a stable indicator established early in adulthood and is less susceptible to reverse causation by health status than occupational position, it was selected as the primary socioeconomic indicator for the multivariable regression analyses. Occupational social class was used mainly to characterise the study population and to examine descriptive socioeconomic gradients.

Occupational category was coded from participants’ usual job titles and grouped into broader categories reflecting the social and functional structure of employment. For analytic purposes, occupations were further collapsed into manual and non-manual groups, following approaches derived from modified occupational classification frameworks based on the International Standard Classification of Occupations and widely used in occupational epidemiology [39].

### 2.4. Clinical, Anthropometric, and Laboratory Assessment

All examinations were performed by trained occupational health personnel under standardised conditions. Body weight and height were measured with participants barefoot and wearing light clothing, and body mass index (BMI) was calculated as weight in kilograms divided by height in metres squared. Waist circumference was measured using standard anatomical landmarks and interpreted according to internationally accepted recommendations for central adiposity assessment [40,41]. Blood pressure was measured by trained occupational health personnel using an automated OMRON M3 sphygmomanometer (Omron Healthcare, Kyoto, Japan) after participants had been seated and resting for at least five minutes. Three measurements were obtained at one-minute intervals, and the average of the final two readings was used for analysis.

Venous blood samples were obtained after an overnight fast and analysed in certified laboratories. Biochemical measurements included fasting plasma glucose, triglycerides, total cholesterol, high-density lipoprotein cholesterol, and gamma-glutamyl transferase. These parameters were used to derive the predefined cardiometabolic indices.

### 2.5. Cardiometabolic Outcomes

Cardiometabolic risk was evaluated across five prespecified domains.

Obesity was defined as BMI ≥ 30 kg/m^2^, according to established adult classification criteria [40].

Probable steatotic liver disease was assessed using the Fatty Liver Index (FLI). Participants with FLI values ≥ 60 were considered to have a high probability of steatotic liver disease in epidemiological terms. Because imaging studies or liver biopsy were not available, FLI ≥ 60 was used as an epidemiological proxy and should not be interpreted as a clinical diagnosis of MASLD [42].

Insulin resistance was primarily defined using the triglyceride-glucose (TyG) index, with values ≥ 8.8 considered indicative of insulin resistance. The metabolic score for insulin resistance (METS-IR) was also calculated as a complementary marker of insulin resistance; however, the binary insulin resistance outcome used in all tables and regression analyses was defined exclusively according to the TyG threshold. Both indices have shown good performance in population-based settings where direct clamp-based assessment is not feasible [43,44].

Atherogenic risk was assessed through lipid-based indices reflecting the balance between pro-atherogenic and anti-atherogenic lipoprotein fractions. The atherogenic index of plasma (AIP) was calculated as the logarithm of the triglyceride-to-HDL cholesterol ratio, and values > 0.24 were considered high risk. The total cholesterol/HDL cholesterol ratio was also examined as a complementary lipid marker [45].

Metabolic syndrome was defined according to the American Heart Association/National Heart, Lung, and Blood Institute update of the NCEP ATP III criteria, requiring the presence of at least three of the following abnormalities: increased waist circumference, elevated triglycerides, reduced HDL cholesterol, elevated blood pressure, and impaired fasting glucose [46].

All cardiometabolic outcomes were analysed as binary variables in order to facilitate comparability across regression models and to preserve consistency with the preceding manuscripts from the same research line.

### 2.6. Lifestyle Variables and Additional Covariates

Smoking status was obtained during the clinical interview and categorised as current smoker, former smoker, or never smoker. Physical activity was assessed with the short form of the International Physical Activity Questionnaire (IPAQ-SF), a widely validated instrument for international epidemiological use [47]. Participants were classified according to standard IPAQ thresholds and, for the main adjusted models, were additionally grouped into physically active versus insufficiently active categories.

Dietary quality was evaluated using the 14-item Mediterranean Diet Adherence Screener (MEDAS-14), originally developed in the PREDIMED framework and subsequently validated in Spanish adults. In the main analysis, adherence was dichotomised as low versus adequate according to conventional cut-off values used in previous studies [48].

Age and sex were included as core covariates in all models. Diabetes was included as a clinical covariate because of its strong association with multiple cardiometabolic outcomes and its potential role as a marker of overall metabolic burden. However, because diabetes may also lie on the pathway linking socioeconomic position and cardiometabolic dysfunction, additional sensitivity analyses excluding diabetes from the fully adjusted models were performed to assess the robustness of the findings. Lifestyle variables were treated as explanatory or intermediate variables rather than purely demographic descriptors.

### 2.7. Statistical Analysis

Continuous variables were summarised as mean and standard deviation, whereas categorical variables were expressed as absolute frequencies and percentages. Baseline characteristics were compared across educational categories using analysis of variance for continuous variables and chi-square tests for categorical variables.

The prevalence of each cardiometabolic outcome was estimated according to educational level and occupational category. Tests for linear trend across educational categories were performed by modelling educational attainment as an ordinal variable.

To evaluate the association between socioeconomic position and each cardiometabolic outcome, separate multivariable logistic regression models were fitted for obesity, probable steatotic liver disease (FLI ≥ 60), insulin resistance, atherogenic risk, and metabolic syndrome. Odds ratios (ORs) and 95% confidence intervals (95% CIs) were calculated.

A sequential adjustment strategy was applied in order to examine how the association changed after progressive inclusion of demographic, behavioural, and clinical variables:

Model 1: crude model.

Model 2: adjusted for age and sex.

Model 3: additionally adjusted for smoking status and physical activity.

Model 4: additionally adjusted for diabetes.

Additionally, tests for linear trend were performed by modelling educational level as an ordinal variable in the regression analyses.

Given the conceptual focus of the present article, an additional exploratory model was used for downstream metabolic outcomes to assess the extent to which inclusion of obesity attenuated the associations observed for MASLD, insulin resistance, atherogenic risk, and metabolic syndrome. This step was intended to evaluate the extent to which inclusion of obesity was associated with attenuation of the observed associations between socioeconomic disadvantage and downstream cardiometabolic outcomes, rather than as a competing independent endpoint. The percentage attenuation of OR estimates across models was examined descriptively to support interpretation of the sequential analyses. This approach was used to facilitate interpretation of the sequential attenuation analyses.

### 2.8. Mediation Analysis

Exploratory mediation analyses were conducted to examine whether obesity could potentially account for part of the observed association between educational level and downstream cardiometabolic outcomes. Educational level was specified as the exposure variable, obesity (BMI ≥ 30 kg/m^2^) as the mediator, and each cardiometabolic outcome as the dependent variable.

Regression-based mediation models were fitted using the PROCESS macro for SPSS [49] (version 4.2; Andrew F. Hayes, Ohio State University), corresponding to a simple mediation model (Model 4). All mediation models were adjusted for age, sex, smoking status, physical activity, Mediterranean diet adherence, and diabetes, consistent with the fully adjusted regression models.

Indirect effects were estimated using non-parametric bootstrapping with 5000 resamples and bias-corrected 95% confidence intervals. Total effects represented the overall association between educational level and each outcome before inclusion of obesity, whereas direct effects represented the association after accounting for obesity.

Because the outcomes were binary, mediation effects were estimated on the log-odds scale and subsequently exponentiated for presentation as odds ratios.

The mediated proportion was calculated as:

Proportion mediated (%) = [(Total effect − Direct effect)/Total effect] × 100.

Because the mediation models were estimated on the log-odds scale, the proportion mediated was derived from the corresponding regression coefficients (log-ORs) rather than from the exponentiated odds ratios. Therefore, the reported mediation percentages cannot be directly reconstructed from the OR estimates alone.

Because exposure, mediator, and outcomes were measured simultaneously, these analyses were considered exploratory and hypothesis-generating and were not intended to establish a causal mediation pathway.

All analyses were performed using IBM SPSS Statistics version 30.0 (IBM Corp., Armonk, NY, USA). Statistical significance was set at a two-sided *p*-value < 0.05.

Multicollinearity among covariates was assessed using variance inflation factors (VIFs) and tolerance statistics. All VIF values were low (range: 1.025–1.114), and all tolerance values exceeded 0.89, indicating no evidence of problematic multicollinearity among the variables included in the regression models.

## 3. Results

### 3.1. Participant Characteristics

A total of 3108 participants were included in the analysis. The mean age of the study population was 43.9 ± 9.8 years, and 1889 participants (60.8%) were men. Educational attainment was distributed as follows: 1042 participants (33.5%) had higher education, 1126 (36.2%) had intermediate education, and 940 (30.2%) had primary or no formal education. Manual occupations were reported by 1451 participants (46.7%). The overall prevalence of obesity, insulin resistance, probable steatotic liver disease, atherogenic risk, and metabolic syndrome was 15.8%, 23.9%, 19.8%, 17.6%, and 14.6%, respectively. Table 1 summarises the baseline characteristics of the study population according to educational level.

Individuals with lower educational attainment were older, more frequently engaged in manual occupations, and exhibited less favourable lifestyle profiles, including higher smoking prevalence, lower physical activity, and poorer adherence to the Mediterranean diet.

A clear gradient was also observed for anthropometric and metabolic variables, with progressively higher body mass index and higher prevalence of all cardiometabolic outcomes—including obesity, insulin resistance, probable steatotic liver disease (FLI ≥ 60), atherogenic risk, and metabolic syndrome—across decreasing educational categories (all *p* for trend < 0.001).

### 3.2. Prevalence of Cardiometabolic Outcomes

The prevalence of cardiometabolic outcomes across educational categories is presented in Table 2.

A clear and consistent socioeconomic gradient was observed across all cardiometabolic outcomes. The prevalence increased progressively with decreasing educational level for obesity, insulin resistance, probable steatotic liver disease (FLI ≥ 60), atherogenic risk, and metabolic syndrome. Tests for linear trend were statistically significant for all outcomes (*p* for trend < 0.001), indicating a progressive socioeconomic gradient across educational categories.

When stratified by occupational category, individuals in manual occupations also exhibited a higher prevalence of cardiometabolic risk factors, although the gradient was less pronounced compared with educational level.

### 3.3. Association Between Educational Level and Obesity

The association between educational level and cardiometabolic outcomes was assessed using multivariable logistic regression models (Table 3).

In crude analyses, lower educational attainment was associated with higher odds of all cardiometabolic outcomes. Progressive adjustment attenuated these associations, particularly after inclusion of behavioural and clinical variables. In fully adjusted models, the association with obesity remained statistically significant, whereas associations with the remaining cardiometabolic outcomes were substantially attenuated.

### 3.4. Attenuation After Inclusion of Obesity

When obesity was included in the regression models for downstream cardiometabolic outcomes, a marked attenuation of associations was observed.

The inclusion of obesity resulted in a substantial reduction in the odds ratios for probable steatotic liver disease (FLI ≥ 60), insulin resistance, atherogenic risk, and metabolic syndrome, with attenuation percentages ranging from approximately 53% to over 72%, depending on the outcome. These findings were consistent across sensitivity analyses. Sensitivity analyses excluding diabetes from the fully adjusted models yielded materially similar results, indicating that adjustment for diabetes did not substantially influence the observed associations (Supplementary Table S1).

After accounting for obesity, the associations between educational level and these cardiometabolic outcomes were substantially attenuated, suggesting that adiposity may partly explain some of the observed relationships between socioeconomic position and metabolic dysfunction. However, because all variables were measured simultaneously, these findings should not be interpreted as evidence of a causal pathway.

To further explore the potential role of obesity as a possible explanatory factor, additional models including obesity were fitted for downstream cardiometabolic outcomes (Table 4).

The inclusion of obesity in the regression models resulted in a substantial attenuation of the associations between educational level and cardiometabolic outcomes. The magnitude of attenuation ranged from approximately 53% to 72%, depending on the outcome.

After accounting for obesity, the associations were no longer statistically significant for any of the downstream cardiometabolic domains. These findings indicate that adjustment for obesity was associated with substantial attenuation of the observed socioeconomic gradients in cardiometabolic risk.

**Figure 2:**
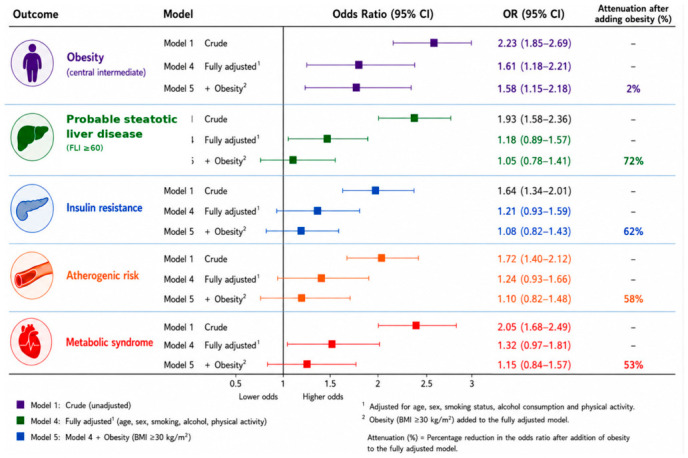
Forest plot of the associations between lower educational attainment and cardiometabolic outcomes before and after adjustment for obesity.

The progressive attenuation of the associations after inclusion of obesity is illustrated in Figure 2.

Forest plot showing odds ratios (ORs) and 95% confidence intervals (95% CIs) for the association between primary/no education versus higher education (reference category) and cardiometabolic outcomes across sequential regression models. Model 1: crude model. Model 4: adjusted for age, sex, smoking status, physical activity and diabetes. Model 5: Model 4 additionally adjusted for obesity (BMI ≥ 30 kg/m^2^). Attenuation (%) represents the percentage reduction in the excess odds ratio after inclusion of obesity in the fully adjusted model. Abbreviations: OR, odds ratio; CI, confidence interval; BMI, body mass index; FLI, Fatty Liver Index.

### 3.5. Mediation Analysis

To further explore the potential role of obesity as a possible contributor to the observed associations, mediation analyses were conducted (Table 5).

Exploratory mediation analyses suggested that obesity may account for part of the observed association between educational level and estimated steatotic liver disease risk, representing approximately 28% of the total association. A smaller proportion of the association with insulin resistance (approximately 8%) was also statistically compatible with an indirect effect through obesity. However, mediation analyses rely on assumptions regarding the temporal ordering of exposure, mediator, and outcome that cannot be verified in a cross-sectional design. Therefore, these findings should be interpreted as a statistical decomposition of the observed associations and considered hypothesis-generating rather than evidence of a confirmed causal mediating relationship.

These findings are compatible with the hypothesis that adiposity may be associated with the observed relationship between socioeconomic position and hepatic metabolic dysfunction, while additional mechanisms are likely to contribute to other cardiometabolic domains.

### 3.6. Additional Analyses

In sex-stratified analyses, the association between lower educational level and obesity appeared more pronounced in men than in women, although the direction of the associations was consistent across both sexes.

No statistically significant interaction between educational level and sex was observed for any of the cardiometabolic outcomes (*p* for interaction > 0.05).

## 4. Discussion

### 4.1. Principal Findings

The present study provides a comprehensive evaluation of the association between socioeconomic position and cardiometabolic risk across multiple metabolic domains. The findings demonstrate a consistent socioeconomic gradient across all cardiometabolic domains, with lower educational attainment associated with a less favourable cardiometabolic profile.

However, when accounting for behavioural and clinical factors, and particularly after the inclusion of obesity in the models, the associations with most downstream cardiometabolic outcomes were substantially attenuated. In contrast, the association between educational level and obesity remained robust across all models. Furthermore, mediation analyses indicated that obesity accounted for a meaningful proportion of the association with probable steatotic liver disease (FLI ≥ 60) and, to a lesser extent, with insulin resistance.

Taken together, these findings suggest that obesity may represent an important explanatory factor associated with attenuation of the observed socioeconomic gradients in cardiometabolic risk, rather than simply an isolated metabolic condition. However, the cross-sectional design does not permit confirmation that obesity lies on the causal pathway between socioeconomic disadvantage and downstream metabolic abnormalities.

### 4.2. Comparison with Previous Literature

The observed socioeconomic gradient in cardiometabolic risk is consistent with a large body of epidemiological evidence showing that individuals with lower socioeconomic position experience a disproportionate burden of metabolic disorders [50,51,52]. Previous studies have reported similar associations for obesity, type 2 diabetes, and cardiovascular disease across diverse populations and healthcare systems, reinforcing the notion that these inequalities are structurally embedded rather than solely driven by individual behaviours [53,54].

In line with our findings, educational attainment has been identified as a particularly strong and stable indicator of socioeconomic position in relation to metabolic health [55,56]. Lower educational levels have been associated with higher prevalence of obesity and adverse metabolic profiles, even after adjustment for lifestyle factors, suggesting the involvement of additional pathways beyond behavioural risk factors [57,58,59].

The role of obesity as a potential explanatory factor in the relationship between socioeconomic position and cardiometabolic outcomes has been increasingly recognised. Several studies have suggested that excess adiposity may contribute to the association between upstream social determinants and downstream metabolic alterations, including insulin resistance and hepatic steatosis [60,61,62]. Our results extend this evidence by quantifying the extent to which adjustment for obesity attenuates these associations within a multidimensional framework, highlighting a particularly relevant association with probable steatotic liver disease (FLI ≥ 60). Given the cross-sectional design, these analyses should be considered exploratory and hypothesis-generating rather than confirmatory evidence of mediation.

At the same time, the attenuation of associations for other cardiometabolic outcomes after adjustment for obesity suggests that not all metabolic domains are equally influenced by this pathway. This is consistent with emerging evidence indicating that cardiometabolic risk is shaped by multiple overlapping mechanisms, including inflammation, hormonal regulation, and environmental exposures, which may operate independently or interactively [63,64].

### 4.3. Potential Mechanisms

Several mechanisms may underlie the observed association between obesity and socioeconomic gradients in cardiometabolic risk. Individuals with lower educational attainment are more likely to be exposed to obesogenic environments characterised by limited access to healthy foods, reduced opportunities for physical activity, and higher levels of chronic stress [65,66]. Chronic psychosocial stress associated with socioeconomic disadvantage may also promote metabolic dysregulation through neuroendocrine pathways, including activation of the hypothalamic–pituitary–adrenal axis and increased cortisol secretion [67]. These mechanisms may contribute to excess adiposity and adverse metabolic profiles, although the present findings cannot determine the temporal sequence of these relationships. From a behavioral perspective, individuals with lower educational attainment are more likely to be exposed to obesogenic environments characterised by limited access to healthy foods, reduced opportunities for physical activity, and higher levels of chronic stress [65,66]. These factors contribute to positive energy balance and long-term weight gain.

In addition, chronic psychosocial stress associated with socioeconomic disadvantage may promote metabolic dysregulation through neuroendocrine pathways, including activation of the hypothalamic–pituitary–adrenal axis and increased cortisol secretion [67]. These processes are known to favour visceral fat accumulation and insulin resistance, thereby amplifying cardiometabolic risk.

Biologically, excess adiposity is strongly associated with systemic inflammation, lipid abnormalities, and ectopic fat deposition, particularly in the liver, which may explain the greater attenuation observed for probable steatotic liver disease (FLI ≥ 60) in our study [68,69]. The clustering of these metabolic alterations further supports the notion that obesity is closely interconnected with multiple cardiometabolic processes, although our findings do not establish a causal mediating role.

### 4.4. Clinical and Public Health Implications

The findings of this study have important implications for both clinical practice and public health strategies. First, they suggest that interventions aimed at reducing cardiometabolic inequalities should prioritise the prevention and management of obesity, particularly among socioeconomically disadvantaged groups.

Second, the results highlight the limitations of approaches focused exclusively on individual lifestyle modification, as these may fail to address the broader structural determinants of obesity. Policies targeting food environments, urban planning, education, and social inequalities are likely to be necessary to achieve meaningful reductions in cardiometabolic risk at the population level [70,71].

Finally, the observation that obesity was associated with attenuation of several downstream cardiometabolic associations reinforces the need for integrated approaches addressing multiple components of metabolic risk, rather than focusing on individual risk factors in isolation. Longitudinal studies are required to determine whether obesity truly mediates these relationships over time.

### 4.5. Strengths and Limitations

This study has several strengths. It is based on a relatively large and well-characterised population of working-age adults, with standardised assessment of sociodemographic, behavioural, and metabolic variables. The use of multiple cardiometabolic outcomes allows for a more comprehensive evaluation of metabolic health compared with single-outcome approaches. In addition, the sequential modelling strategy and mediation analysis provide insight into potential pathways underlying the observed associations.

However, several limitations should be acknowledged. The cross-sectional design precludes causal inference, and the observed mediation effects should be interpreted with caution. Furthermore, mediation analyses assume a temporal ordering between exposure, mediator, and outcome that cannot be verified in the present cross-sectional study.

Another important limitation is the potential mathematical overlap between obesity and some of the analysed cardiometabolic outcomes. The Fatty Liver Index incorporates body mass index and waist circumference, METS-IR includes body mass index in its calculation, and metabolic syndrome includes central obesity as one of its diagnostic components. Therefore, part of the attenuation observed after adjustment for obesity may reflect shared or overlapping components between the exposure and outcome definitions rather than a true biological mediating process. Consequently, these findings should be interpreted cautiously and regarded as hypothesis-generating.

Socioeconomic position was assessed using educational level and occupational category, which, although widely used, may not fully capture all dimensions of social disadvantage. Residual confounding cannot be excluded, particularly for unmeasured factors such as income, early-life conditions, or environmental exposures. Finally, the use of surrogate markers for some cardiometabolic outcomes, although validated in epidemiological settings, may introduce some degree of misclassification.

Although alcohol consumption was considered in the analyses, information regarding chronic liver disease, viral hepatitis, and hepatotoxic medication use was not available. Consequently, residual confounding in the analyses of FLI-defined probable steatotic liver disease cannot be completely excluded.

In addition, information regarding the use of antihypertensive, lipid-lowering, and glucose-lowering medications was not available. Because these therapies may influence several cardiometabolic outcomes and risk estimates, residual confounding related to medication use cannot be completely excluded.

In addition, the study population consisted exclusively of working-age adults participating in routine occupational health surveillance programmes. Consequently, unemployed individuals, retired adults, persons with severe illness, and those outside formal employment were not represented. This may have introduced a healthy worker effect and occupational selection bias, potentially leading to an underestimation or distortion of the true socioeconomic gradients in cardiometabolic risk. Therefore, caution is warranted when generalising these findings to the broader adult population.

Although both educational attainment and occupational social class were assessed, the multivariable analyses focused primarily on education because it is generally considered a more stable indicator of socioeconomic position across the life course and less prone to health-related occupational selection. Consequently, potential associations specific to occupational social class may not have been fully captured.

Because several cardiometabolic outcomes were relatively common, odds ratios may have overestimated the magnitude of some associations. Future studies could consider alternative approaches, such as robust Poisson regression and prevalence ratios, as complementary sensitivity analyses.

## 5. Conclusions

In conclusion, this study demonstrates that socioeconomic inequalities in cardiometabolic risk are strongly patterned across multiple metabolic domains. Obesity emerged as the most consistent and robustly associated outcome and was associated with substantial attenuation of several downstream cardiometabolic associations, particularly for probable steatotic liver disease (FLI ≥ 60). These findings are compatible with the hypothesis that obesity may partly explain some of the observed socioeconomic differences in cardiometabolic health, although the cross-sectional design precludes causal interpretation. Addressing obesity within its broader social context may therefore represent a potential strategy to reduce health inequalities among employed working-age adults and may inform preventive strategies in occupational health settings; however, longitudinal studies are needed to clarify the temporal and causal relationships underlying these associations.

## Figures and Tables

**Table 1:** Baseline characteristics of the study population according to educational level.

Variable	Higher Education (*n* = 1042)	Intermediate Education (*n* = 1126)	Primary/No Education (*n* = 940)	*p*-Value
Age (years), mean (SD)	41.2 (9.3)	43.8 (9.7)	46.5 (10.2)	<0.001
Male sex, *n* (%)	612 (58.7)	676 (60.0)	601 (63.9)	0.041
Manual occupation, *n* (%)	318 (30.5)	521 (46.3)	612 (65.1)	<0.001
Current smokers, *n* (%)	248 (23.8)	301 (26.7)	294 (31.3)	<0.001
Physically active, *n* (%)	672 (64.5)	651 (57.8)	472 (50.2)	<0.001
High Mediterranean diet adherence, *n* (%)	598 (57.4)	564 (50.1)	403 (42.9)	<0.001
BMI (kg/m^2^), mean (SD)	25.6 (3.8)	26.8 (4.1)	28.1 (4.6)	<0.001
Obesity, *n* (%)	120 (11.5)	185 (16.4)	186 (19.8)	<0.001
Insulin resistance, *n* (%)	198 (19.0)	274 (24.3)	271 (28.8)	<0.001
Probable steatotic liver disease (FLI ≥ 60), *n* (%)	156 (15.0)	233 (20.7)	225 (23.9)	<0.001
Atherogenic risk, *n* (%)	142 (13.6)	206 (18.3)	198 (21.1)	<0.001
Metabolic syndrome, *n* (%)	108 (10.4)	169 (15.0)	176 (18.7)	<0.001

**Table 2:** Prevalence of cardiometabolic outcomes according to educational level.

Outcome	Higher Education (%)	Intermediate Education (%)	Primary/No Education (%)	*p* for Trend
Obesity	11.5	16.4	19.8	<0.001
Insulin resistance	19.0	24.3	28.8	<0.001
Probable steatotic liver disease (FLI ≥ 60)	15.0	20.7	23.9	<0.001
Atherogenic risk	13.6	18.3	21.1	<0.001
Metabolic syndrome	10.4	15.0	18.7	<0.001

**Table 3:** Association between educational level and cardiometabolic outcomes: multivariable logistic regression models.

Outcome	Educational Level	Model 1 OR (95% CI)	Model 2 OR (95% CI)	Model 3 OR (95% CI)	Model 4 OR (95% CI)
Obesity	Intermediate	1.51 (1.18–1.93)	1.42 (1.10–1.83)	1.36 (1.05–1.76)	1.34 (1.03–1.74)
	Primary/no education	1.89 (1.46–2.45)	1.72 (1.32–2.24)	1.63 (1.24–2.13)	1.61 (1.18–2.21)
Insulin resistance	Intermediate	1.36 (1.08–1.71)	1.28 (1.01–1.62)	1.18 (0.92–1.50)	1.12 (0.87–1.45)
	Primary/no education	1.69 (1.33–2.15)	1.52 (1.18–1.96)	1.34 (1.03–1.74)	1.21 (0.93–1.59)
Probable steatotic liver disease (FLI ≥ 60)	Intermediate	1.33 (1.03–1.71)	1.25 (0.97–1.61)	1.15 (0.89–1.49)	1.10 (0.85–1.43)
	Primary/no education	1.61 (1.24–2.10)	1.45 (1.10–1.90)	1.28 (0.97–1.69)	1.18 (0.89–1.57)
Atherogenic risk	Intermediate	1.39 (1.07–1.80)	1.31 (1.00–1.71)	1.20 (0.92–1.58)	1.15 (0.87–1.52)
	Primary/no education	1.68 (1.28–2.21)	1.50 (1.13–1.98)	1.33 (1.00–1.77)	1.24 (0.93–1.66)
Metabolic syndrome	Intermediate	1.52 (1.16–2.00)	1.41 (1.07–1.86)	1.28 (0.97–1.70)	1.22 (0.92–1.63)
	Primary/no education	1.97 (1.49–2.61)	1.73 (1.29–2.31)	1.48 (1.10–2.00)	1.32 (0.97–1.81)

Model 1: crude. Model 2: adjusted for age and sex. Model 3: additionally adjusted for smoking status and physical activity. Model 4: additionally adjusted for diabetes. Reference category: higher education.

**Table 4:** Attenuation of the association between educational level and cardiometabolic outcomes after inclusion of obesity.

Outcome	OR Model 4 (Without Obesity)	OR Model 5 (with Obesity)	% Attenuation
Insulin resistance	1.21 (0.93–1.59)	1.08 (0.82–1.43)	62%
Probable steatotic liver disease (FLI ≥ 60)	1.18 (0.89–1.57)	1.05 (0.78–1.41)	72%
Atherogenic risk	1.24 (0.93–1.66)	1.10 (0.82–1.48)	58%
Metabolic syndrome	1.32 (0.97–1.81)	1.15 (0.84–1.57)	53%

Model 4: adjusted for age, sex, smoking status, physical activity and diabetes. Model 5: Model 4 additionally adjusted for obesity (BMI ≥ 30 kg/m^2^). Attenuation (%) was calculated as [(OR Model 4 − OR Model 5)/(OR Model 4 − 1)] × 100 and represents the percentage reduction in the excess odds ratio after inclusion of obesity in the model.

**Table 5:** Mediation analysis of the association between educational level and cardiometabolic outcomes through obesity.

Outcome	Total Effect OR (95% CI)	Direct Effect OR (95% CI)	Indirect Effect OR (95% CI)	% Mediated
Probable steatotic liver disease (FLI ≥ 60)	1.18 (0.89–1.57)	1.05 (0.78–1.41)	1.12 (1.05–1.20)	28%
Insulin resistance	1.21 (0.93–1.59)	1.08 (0.82–1.43)	1.08 (1.02–1.14)	8%

Total effect: association between educational level and outcome without inclusion of obesity. Direct effect: association after inclusion of obesity in the model. Indirect effect: effect mediated through obesity. Percentage mediated was calculated using the underlying regression coefficients (log-ORs) derived from the mediation models and therefore cannot be reproduced directly from the exponentiated odds ratios shown in the table.

## Data Availability

The original contributions presented in this study are included in the article/Supplementary Material. Further inquiries can be directed to the corresponding author(s).

## Supplementary Materials

The following supporting information can be downloaded at: https://www.mdpi.com/article/10.3390/medsci14030382/s1, Table S1: Sensitivity analyses excluding diabetes from the fully adjusted models.

## References

[1] Chong B., Jayabaskaran J., Jauhari S.M., Chan S.P., Goh R., Kueh M.T.W., Li H., Chin Y.H., Kong G., Anand V.V. (2025). Global burden of cardiovascular diseases: Projections from 2025 to 2050. Eur. J. Prev. Cardiol..

[2] Vaduganathan M., Mensah G.A., Turco J.V., Fuster V., Roth G.A. (2022). The Global Burden of Cardiovascular Diseases and Risk: A Compass for Future Health. J. Am. Coll. Cardiol..

[3] World Health Organization (2025). Cardiovascular Diseases (CVDs).

[4] Vicente-Herrero M.T., Garrido-Sepúlveda L., Tárraga-López P.J., López-González Á.A., Ramírez-Manent J.I., Garrido J.A. (2025). Impact of the Goldberg scale on cardiometabolic health: A study of 119,336 Spanish workers. Acad. J. Health Sci..

[5] Kang P.S., Neeland I.J. (2023). Body Fat Distribution, Diabetes Mellitus, and Cardiovascular Disease: An Update. Curr. Cardiol. Rep..

[6] GBD 2021 Adult BMI Collaborators (2025). Global, regional, and national prevalence of adult overweight and obesity, 1990–2021, with forecasts to 2050: A forecasting study for the Global Burden of Disease Study 2021. Lancet.

[7] Hruby A., Manson J.E., Qi L., Malik V.S., Rimm E.B., Sun Q., Willett W.C., Hu F.B. (2016). Determinants and Consequences of Obesity. Am. J. Public Health.

[8] Stringhini S., Carmeli C., Jokela M., Muennig P., Guida F., Ricceri F., D’ERrico A., Barros H., Bochud M., Chadeau-Hyam M. (2017). Socioeconomic status and the 25 × 25 risk factors as determinants of premature mortality: A multicohort study and meta-analysis of 1·7 million men and women. Lancet.

[9] Hao Z., Wang M., Zhu Q., Li J., Liu Z., Yuan L., Zhang Y., Zhang L. (2022). Association Between Socioeconomic Status and Prevalence of Cardio-Metabolic Risk Factors: A Cross-Sectional Study on Residents in North China. Front. Cardiovasc. Med..

[10] Walsh D., Hoehn A., Dundas R., McCartney G., Whyte B. (2026). Using lifespan variation to better understand long-term trends in health inequalities in Scotland and Europe. Eur. J. Public Health.

[11] Zhou L., Nutakor J.A., Larnyo E., Addai-Dansoh S., Cui Y., Gavu A.K., Kissi J. (2024). Exploring socioeconomic status, lifestyle factors, and cardiometabolic disease outcomes in the United States: Insights from a population-based cross-sectional study. BMC Public Health.

[12] Aguiló Juanola M.C., López-González A.A., Tomás-Gil P., Paublini H., Tárraga-López P.J., Ramírez-Manent J.I. (2024). Influence of tobacco consumption on the values of different cardiometabolic risk scales in 418,343 spanish workers. Acad. J. Health Sci..

[13] Morelli V. (2023). Social Determinants of Health: An Overview for the Primary Care Provider. Prim. Care.

[14] Borkowski P., Borkowska N., Mangeshkar S., Adal B.H., Singh N. (2024). Racial and Socioeconomic Determinants of Cardiovascular Health: A Comprehensive Review. Cureus.

[15] Hanlon P., Politis M., Wightman H., Kirkpatrick S., Jones C., Khan M., Bezzina C., Mackinnon S., Rennison H., Wei L. (2024). Frailty and socioeconomic position: A systematic review of observational studies. Ageing Res. Rev..

[16] Nie P., Ding L., Sousa-Poza A., Leon A.A., Xue H., Jia P., Wang L., Sánchez M.E.D., Wang Y. (2020). Socioeconomic position and the health gradient in Cuba: Dimensions and mechanisms. BMC Public Health.

[17] Curtin E.L., Widnall E., Dodd S., Limmer M., Simmonds R., Russell A.E., Kaley A., Kidger J. (2024). Exploring mechanisms and contexts in a Peer Education Project to improve mental health literacy in schools in England: A qualitative realist evaluation. Health Educ. Res..

[18] Tárraga Marcos P.J., López-González Á.A., Martínez-Almoyna Rifá E., Paublini Oliveira H., Martorell Sánchez C., Tárraga López P.J., Ramírez-Manent J.I. (2025). Variables associated with overweight and obesity in Spanish healthcare workers. Acad. J. Health Sci..

[19] Marina Arroyo M., Ramírez Gallegos I., López-González A.A., Vicente-Herrero M.T., Vallejos D., Tárraga López P.J., Ramírez Manent J.I. (2024). Equation Córdoba body fat values according to sociodemographic variables and healthy habits in 386,924 Spanish workers. Acad. J. Health Sci..

[20] Mestre-Font M., Busquets-Cortés C., Ramírez-Manent J.I., Tomás-Gil P., Paublini H., López-González A.A. (2024). Influence of sociodemographic variables and healthy habits on the values of overweight and obesity scales in 386,924 Spanish workers. Acad. J. Health Sci..

[21] Boylan J.M., Cundiff J.M., Jakubowski K.P., Pardini D.A., Matthews K.A. (2018). Pathways Linking Childhood SES and Adult Health Behaviors and Psychological Resources in Black and White Men. Ann. Behav. Med..

[22] The Lancet (2024). Health equity in Ireland: Past, present, and future. Lancet.

[23] López-González Á.A., Villarroel G., Riveras Capote A., Camero-Ávalos Á. (2025). Factores de riesgo cardiometabólico en 37.936 trabajadores de la salud españoles. Acad. J. Health Sci..

[24] Lin X., Li H. (2021). Obesity: Epidemiology, Pathophysiology, and Therapeutics. Front. Endocrinol..

[25] Petersen M.C., Smith G.I., Palacios H.H., Farabi S.S., Yoshino M., Yoshino J., Cho K., Davila-Roman V.G., Shankaran M., Barve R.A. (2024). Cardiometabolic characteristics of people with metabolically healthy and unhealthy obesity. Cell Metab..

[26] Devaux M., Sassi F. (2013). Social inequalities in obesity and overweight in 11 OECD countries. Eur. J. Public Health.

[27] Liu J., Fang Z., Lu Q., Wang Y., Zhang L. (2025). Projecting Global Trends and Inequalities in Adult Overweight and Obesity, 2023–2040: Findings from the NCD-RisC Database. Obesity.

[28] García-Samuelsson M., Tárraga-López P.J., López-González Á.A., Busquets-Cortés C., Ramírez-Gallegos A., Obrador de Hevia J., Ramírez-Manent J.I. (2025). Comparative evolution of metabolic health status in obese individuals using novel and conventional anthropometric indexes: A decade-long analysis in Spanish workers. Acad. J. Health Sci..

[29] Fernández-Figares Vicioso M.P., del Barrio Fernández J.L., López-González A.A., Ramírez-Manent J.I., Vicente Herrero M.T. (2024). Prevalencia de factores de riesgo cardiometabólico. Comparativa sector Comercio vs. Industria y variables asociadas. Acad. J. Health Sci..

[30] Tan M.Y., Zhang Y.J., Zhu S.X., Wu S., Zhang P., Gao M. (2025). The prognostic significance of stress hyperglycemia ratio in evaluating all-cause and cardiovascular mortality risk among individuals across stages 0–3 of cardiovascular–kidney–metabolic syndrome: Evidence from two cohort studies. Cardiovasc. Diabetol..

[31] O’Neill S., O’Driscoll L. (2015). Metabolic syndrome: A closer look at the growing epidemic and its associated pathologies. Obes. Rev..

[32] Wagner C., Carmeli C., Jackisch J., Kivimäki M., van der Linden B.W.A., Cullati S., Chiolero A. (2024). Life course epidemiology and public health. Lancet Public Health.

[33] Kendig H., Gong C.H., Yiengprugsawan V., Silverstein M., Nazroo J. (2017). Life course influences on later life health in China: Childhood health exposure and socioeconomic mediators during adulthood. SSM Popul. Health.

[34] Hoffman D.J., Powell T.L., Barrett E.S., Hardy D.B. (2021). Developmental origins of metabolic diseases. Physiol. Rev..

[35] Arima Y., Fukuoka H. (2020). Developmental origins of health and disease theory in cardiology. J. Cardiol..

[36] Chaturvedi A., Zhu A., Gadela N.V., Prabhakaran D., Jafar T.H. (2024). Social Determinants of Health and Disparities in Hypertension and Cardiovascular Diseases. Hypertension.

[37] Zhao Y. (2023). Socioeconomic Positions and Midlife Health Trajectories in a Changing Social Context: Evidence from China, 1991–2006. J. Health Soc. Behav..

[38] von Elm E., Altman D.G., Egger M., Pocock S.J., Gøtzsche P.C., Vandenbroucke J.P., STROBE Initiative (2007). The Strengthening the Reporting of Observational Studies in Epidemiology (STROBE) Statement: Guidelines for Reporting Observational Studies. Lancet.

[39] Choi S.B., Yoon J.H., Lee W. (2020). The Modified International Standard Classification of Occupations defined by the clustering of occupational characteristics in the Korean Working Conditions Survey. Ind. Health.

[40] (1998). Clinical guidelines on the identification, evaluation, and treatment of overweight and obesity in adults: Executive summary: Expert Panel on the Identification, Evaluation, and Treatment of Overweight in Adults. Am. J. Clin. Nutr..

[41] Nishida C., Ko G.T., Kumanyika S. (2010). Body fat distribution and noncommunicable diseases in populations: Overview of the 2008 WHO Expert Consultation on Waist Circumference and Waist-Hip Ratio. Eur. J. Clin. Nutr..

[42] Recatalá Gimeno M.J., Pérez Lema M. (2024). Determination of the risk of fatty liver and hepatic fibrosis in the working population of the Balearic Islands. Acad. J. Health Sci..

[43] Sun Y., Ji H., Sun W., An X., Lian F. (2025). Triglyceride glucose (TyG) index: A promising biomarker for diagnosis and treatment of different diseases. Eur. J. Intern Med..

[44] Duan M., Zhao X., Li S., Miao G., Bai L., Zhang Q., Yang W., Zhao X. (2024). Metabolic score for insulin resistance (METS-IR) predicts all-cause and cardiovascular mortality in the general population: Evidence from NHANES 2001–2018. Cardiovasc. Diabetol..

[45] Zheng G., Jin J., Wang F., Zheng Q., Shao J., Yao J., Huang P., Zhou H., Zhou J. (2025). Association between atherogenic index of plasma and future risk of cardiovascular disease in individuals with cardiovascular-kidney-metabolic syndrome stages 0–3: A nationwide prospective cohort study. Cardiovasc. Diabetol..

[46] Vicente-Herrero M.T., Gordito Soler M., García Agudo S., Vallejos D., López-González A.A., Ramírez-Manent J.I. (2024). Cardiometabolic risk level in 1136 Spanish professional musicians. Acad. J. Health Sci..

[47] Sember V., Meh K., Sorić M., Starc G., Rocha P., Jurak G. (2020). Validity and Reliability of International Physical Activity Questionnaires for Adults across EU Countries: Systematic Review and Meta Analysis. Int. J. Environ. Res. Public Health.

[48] Celada Roldán C., López Díez J., Rider F., Cerezuela Abarca M.Á., Tárraga Marcos A., López P.J.T. (2025). Evaluation of adherence to the Mediterranean diet after a nutritional dietary intervention from primary care. Nutr. Hosp..

[49] Hayes A.F. (2022). Introduction to Mediation, Moderation, and Conditional Process Analysis: A Regression-Based Approach.

[50] NCD Risk Factor Collaboration (NCD-RisC) (2016). Trends in adult body-mass index in 200 countries from 1975 to 2014: A pooled analysis of 1698 population-based measurement studies with 19·2 million participants. Lancet.

[51] Kivimäki M., Batty G.D., Pentti J., Shipley M.J., Sipilä P., Nyberg S.T., Suominen S.B., Oksanen T., Stenholm S., Virtanen M. (2020). Association between socioeconomic status and the development of mental and physical health conditions in adulthood: A multi-cohort study. Lancet Public Health.

[52] Paudel S., Ahmadi M., Phongsavan P., Hamer M., Stamatakis E. (2023). Do associations of physical activity and sedentary behaviour with cardiovascular disease and mortality differ across socioeconomic groups? A prospective analysis of device-measured and self-reported UK Biobank data. Br. J. Sports Med..

[53] Kaur G., Masket D., Reddy T., Revankar S., Satish P., Paquin A., Mulvagh S., O’dOnoghue M.L., Zieroth S., Farkouh M. (2024). Socioeconomic Disparities in Women’s Cardiovascular Health in the United States and Canada. Can. J. Cardiol..

[54] Schultz W.M., Kelli H.M., Lisko J.C., Varghese T., Shen J., Sandesara P., Quyyumi A.A., Taylor H.A., Gulati M., Harold J.G. (2018). Socioeconomic Status and Cardiovascular Outcomes: Challenges and Interventions. Circulation.

[55] Edwards A.C., Lönn S.L., Chartier K.G., Lannoy S., Sundquist J., Kendler K.S., Sundquist K. (2024). Socioeconomic position indicators and risk of alcohol-related medical conditions: A national cohort study from Sweden. PLoS Med..

[56] Dunn J.R. (2010). Health behavior vs the stress of low socioeconomic status and health outcomes. JAMA.

[57] Adamou H., Paquette M.C., François D., Robitaille É., Ouindpanga S.S., Lebel A. (2025). Life-course socioeconomic status and obesity: Scoping review. Popul. Health Metr..

[58] Niu J., Li X., Chen Q., Yang W., Suo L., Chen Z. (2025). Association Between Educational Inequality and Income Inequality with Metabolic Diseases and Cause-Specific Mortality. Clin. Cardiol..

[59] Bridger Staatz C., Kelly Y., Lacey R.E., Blodgett J.M., George A., Arnot M., Walker E., Hardy R. (2021). Life course socioeconomic position and body composition in adulthood: A systematic review and narrative synthesis. Int. J. Obes..

[60] Washington T.B., Johnson V.R., Kendrick K., Ibrahim A.A., Tu L., Sun K., Stanford F.C. (2023). Disparities in Access and Quality of Obesity Care. Gastroenterol. Clin. N. Am..

[61] Hassapidou M., Vlassopoulos A., Kalliostra M., Govers E., Mulrooney H., Ells L., Salas X.R., Muscogiuri G., Darleska T.H., Busetto L. (2023). European Association for the Study of Obesity Position Statement on Medical Nutrition Therapy for the Management of Overweight and Obesity in Adults Developed in Collaboration with the European Federation of the Associations of Dietitians. Obes. Facts..

[62] Anekwe C.V., Jarrell A.R., Townsend M.J., Gaudier G.I., Hiserodt J.M., Stanford F.C. (2020). Socioeconomics of Obesity. Curr. Obes. Rep..

[63] Tosoratto J., Carriedo B., Cantón C. (2024). Cardiometabolic risk level in 43,074 Spanish office workers: Associated variables. Acad. J. Health Sci..

[64] Valenzuela P.L., Carrera-Bastos P., Castillo-García A., Lieberman D.E., Santos-Lozano A., Lucia A. (2023). Obesity and the risk of cardiometabolic diseases. Nat. Rev. Cardiol..

[65] Dietz W.H., Pryor S. (2022). How Can We Act to Mitigate the Global Syndemic of Obesity, Undernutrition, and Climate Change?. Curr. Obes. Rep..

[66] Malo J.S., Schafer M.H., Stull A.J. (2025). Healthy eating in life course context: Asymmetric implications of socioeconomic origins and destinations. Soc. Sci. Med..

[67] Młynarska E., Czarnik W., Dzieża N., Jędraszak W., Majchrowicz G., Prusinowski F., Stabrawa M., Rysz J., Franczyk B. (2025). Type 2 Diabetes Mellitus: New Pathogenetic Mechanisms, Treatment and the Most Important Complications. Int. J. Mol. Sci..

[68] Hammerich L., Tacke F. (2023). Hepatic inflammatory responses in liver fibrosis. Nat. Rev. Gastroenterol. Hepatol..

[69] Younossi Z.M., Golabi P., Paik J.M., Henry A., Van Dongen C., Henry L. (2023). The global epidemiology of nonalcoholic fatty liver disease (NAFLD) and nonalcoholic steatohepatitis (NASH): A systematic review. Hepatology.

[70] Marmot M., Allen J., Goldblatt P., Boyce T., McNeish D., Grady M., Geddes I., Der G., Feeney C., Taylor J. (2010). Fair Society, Healthy Lives.

[71] McGowan V.J., Bambra C. (2022). COVID-19 mortality and deprivation: Pandemic, syndemic, and endemic health inequalities. Lancet Public Health.

