# Supplementary material for: Obesity as a Potential Explanatory Factor in the Association Between Socioeconomic Position and Multidimensional Cardiometabolic Risk
Source: Med Sci (Basel). 2026 Jul 9;14(3):382. doi: 10.3390/medsci14030382 (PMC13413732; doi:10.3390/medsci14030382)
Supplement: Supplementary file 1 [file medsci-14-00382-s001.zip › medsci-4388118-supplementary.pdf]

Supplementary Table S1. Sensitivity analyses excluding diabetes from the fully adjusted models.

Outcome	Educational level	OR (95% CI)
**Obesity**	Intermediate education	1.01 (0.73–1.40)
	Primary/no education	1.50 (1.14–1.99)
**Insulin resistance**	Intermediate education	1.34 (0.96–1.85)
	Primary/no education	1.46 (1.09–1.96)
**Probable steatotic liver disease (FLI ≥60)**	Intermediate education	1.39 (1.05–1.83)
	Primary/no education	1.88 (1.47–2.40)
**Atherogenic risk**	Intermediate education	0.79 (0.62–1.01)
	Primary/no education	0.84 (0.68–1.04)
**Metabolic syndrome**	Intermediate education	1.05 (0.76–1.45)
	Primary/no education	1.13 (0.85–1.51)

Footnote: Odds ratios were adjusted for age, sex, smoking status, and physical activity, excluding diabetes from the fully adjusted models. Higher education was used as the reference category.
